# Key Genomic Regions of Rice Cultivar GuiHeFeng and Its Derivatives Revealed by Genome-Wide Analysis

**DOI:** 10.3390/plants15030520

**Published:** 2026-02-06

**Authors:** Yu-Zhi Chen, Xin-Yu Hao, Yue-Xiong Zhang, Zeng-Feng Ma, Chi Liu, Xiao-Long Zhou, Min-Yi Wei, Bao-Xiang Qin, Yong Yan, Da-Hui Huang

**Affiliations:** 1 Rice Research Institute, Guangxi Academy of Agricultural Sciences, Nanning 530007, China; 2 Guangxi Key Laboratory of Rice Genetics and Breeding, Nanning 530007, China; 3 College of Agriculture, Guangxi University, Nanning 530004, China; 4 Microbiology Research Institute, Guangxi Academy of Agricultural Sciences, Nanning 530007, China

**Keywords:** rice, genome resequencing, single-nucleotide polymorphisms, gene, breeding

## Abstract

Rice is a widely cultivated staple crop that serves as the primary source of carbohydrates for more than half of the global population. Elite parents with superior agronomic traits play a crucial role in rice breeding systems. In this study, we performed whole-genome resequencing of the rice cultivar GuiHeFeng and its nine derivative lines, identifying a total of 6,633,507 high-quality single-nucleotide polymorphisms (SNPs). The percentage of GuiHeFeng traceable blocks (GTBs) in the nine derivatives ranged from 48.94% to 63.2%. Based on the SNP analysis, we found 1310 key GuiHeFeng traceable blocks (kGTBs), which were derived from GuiHeFeng and present in all nine derivatives. Moreover, 375 selective sweeps (SSWs) were identified, of which 20 were also located within the kGTBs. These 20 SSWs were regarded as key genomic regions (kGRs) for rice breeding. After the association test, 20 alleles including 17 genes were identified on the kGTBs, and 38 significant genes were found within the key genomic regions. This research identifies the genomic segments and agronomically important genes/QTLs that will serve as essential targets for genomic selection in rice breeding.

## 1. Introduction

Rice (*Oryza sativa* L.) is a vital food crop and the primary staple for over half of the global population [1]. It serves as a fundamental pillar of worldwide food security [2]. To address the rising food requirements of an expanding global populace, rice output has seen consistent growth in recent decades, largely attributed to the creation of new high-yielding varieties [3]. The introduction of semi-dwarf cultivars, a central element of the initial Green Revolution, led to a substantial boost in rice productivity during the 1960s. Hybrid rice varieties have further enhanced production by 9% over conventional types [4]. Nowadays, rice production confronts challenges of rapid population growth, shrinking farmland, climate change and pest/disease pressure [5]. To ensure worldwide food security, there is an urgent need to create new rice varieties that offer higher yields and greater resilience to both biotic and abiotic stresses. Traditional breeding remains inefficient for developing new varieties due to limited understanding of genetic mechanisms and the time-consuming, labor-intensive process of selecting the target traits [6]. The brand-new concept of 5G breeding, genomic breeding (GB), which encompasses marker-assisted selection (MAS) and genomic selection (GS), appears to be a highly effective strategy for producing new high-yielding rice varieties capable of withstanding stressful conditions and unpredictable climate shifts [6].

Several key characteristics of rice are governed by genes or quantitative trait loci (QTLs) with substantial effects. Marker-assisted selection (MAS) for major-effect genes/QTLs has been widely applied to improve agronomic traits, such as yield, disease resistance, and stress tolerance. However, most agronomic traits are influenced by QTLs with minor phenotypic contributions [7]. Minor-effect QTLs are hampered from being utilized in marker-assisted selection (MAS) mainly because of their uncertain role in different genetic backgrounds and growing environments [8]. It is necessary to identify a robust consensus genomic region for minor-effect QTLs to improve their effectiveness in MAS [9,10]. Analyses of the key/conserved regions that contain the excellent alleles in elite germplasms and the foundation parents is a good alternative for identifying these consensus genomic regions relevant to important agronomic traits [11]. Identifying the key genomic regions is fundamental to understanding the genetic basis of elite traits and accelerating the breeding of improved crop varieties [12,13,14].

Backbone parents, which carry accumulated beneficial agronomic traits, such as disease resistance, high yield, and adaptability, have played a crucial role in modern crop breeding programs due to their ability to transmit desirable traits to offspring through selective breeding [15,16,17]. These parents are foundational in crop breeding, as evidenced by their widespread use in major Chinese rice varieties (e.g., 70% derived from 35 backbone parents between 1950 and 2008) [18]. A large number of superior alleles have been gathered and distributed on different genomic regions due to selective sweeps pyramiding over long-time pedigree breeding progress of backbone parents. Through large-scale genome sequencing in combination with pedigree analysis, some key genomic regions, which can stably inherit in different genetic backgrounds of the pedigree, have been found in rice backbone parents, such as Minghui63, Huanghuazhan, Shuhui527 and Jiayu253 [5,18,19,20]. These key genomic regions are important for genomic selection, such as genome-wide marker-assisted selection, to develop new rice cultivars [11]. These four backbone parents were developed or released more than two decades ago, in 1980, 1996, 2005 and 2005, for Minghui 63, Shuhui 527, Huanghuazhan and Jiayu 253, respectively. Nevertheless, the genomic structure of rice cultivars will continue to evolve due to shifts in their growing conditions and production objectives [6]. Only a few rice cultivars have been analyzed through genome sequencing to identify the critical genomic regions associated with important traits. Moreover, little is known about the key genomic regions architecture of the rice varieties developed in recent years.

GuiHeFeng is an elite conventional rice cultivar released in 2015. In the Guangxi regional yield trials, it demonstrated a significant yield increase of 12.32% compared to the standard control cultivar LiuShaYouZhan202 (the widely recognized benchmark for high-quality conventional rice in Guangxi), meeting the criteria for being classified as “high-yielding” in the context of official varietal release standards. More than 10 excellent cultivars have been derived from GuiHeFeng. In this study, GuiHeFeng and its nine derivatives were selected for whole-genome resequencing (Table 1). Using this sequence information, we were able to uncover the key genomic regions of GuiHeFeng conserved in all its derivatives. We further analyzed the known loci related to important rice traits or unknown QTLs by an association analysis, revealing the basis for the excellent performance of GuiHeFeng and all its derivatives. This comprehensive study of genomic architecture of GuiHeFeng and its derivatives will provide the key genomic regions and important agronomic genes/QTLs for rice high-yield breeding by genomic selection (GS).

## 2. Results

### 2.1. The Derivatives Exhibited Comparable Agronomic Trait Performance to GuiHeFeng

**Figure 1:**
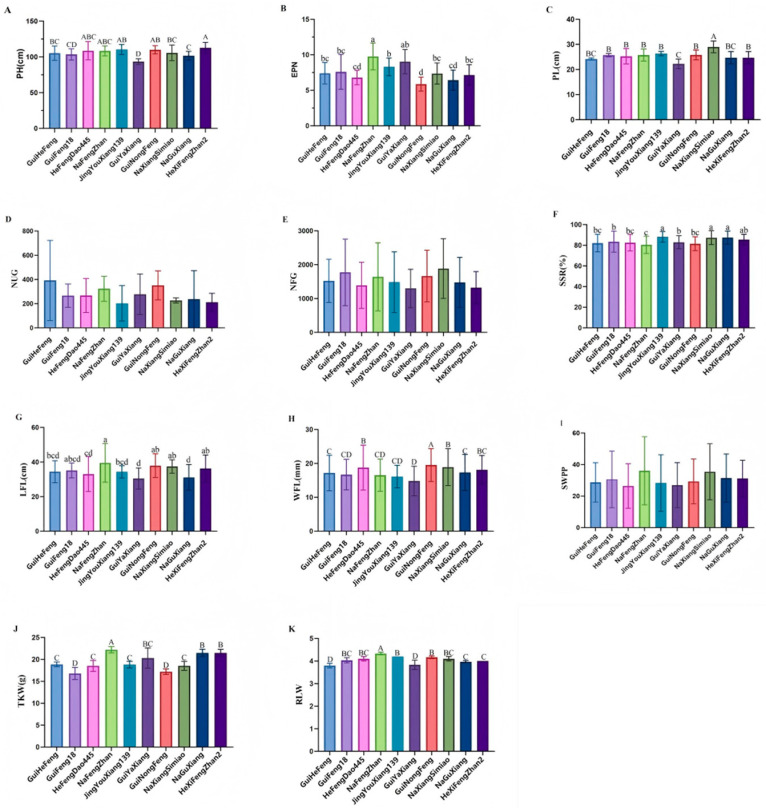
Agronomic traits. (**A**) Plant height (PH). (**B**) Effective panicle number (EPN). (**C**) Panicle length (PL). (**D**) Number of unfilled grains (NUG). (**E**) Number of filled grains (NFG). (**F**) Seed setting rate (SSR). (**G**) Length of flag leaf (LFL). (**H**) Width of flag leaf (WFL). (**I**) Seed weight per plant (SWPP). (**J**) Thousand-kernel weight (TKW). (**K**) Ratio of length and width (RLW) was investigated during all growth seasons. Statistical analysis was performed using SPSS software (version27.0, IBM-Corp., Armonk, NY, USA). The least significant difference LSD test was used for multiple comparisons in the same comparison group, values with different P small letter superscripts mean a significant difference (*p* < 0.05), and different capital letter superscripts mean a very significant difference (*p* < 0.01).

Investigation of 11 agronomic traits was conducted for all the cultivars (Figure 1 and Table S1). Only the plant height of HeXiFengZhan2 and GuiYaXiang was higher and lower than that of GuiHeFeng at significant levels, respectively, and there was no significant difference in plant height between GuiHeFeng and the other seven cultivars (Figure 1A). The effective panicle number (EPN) of NaFengZhan and GuiYaXiang was significantly higher than that of GuiHeFeng, and the EPN of GuiNongFeng was significantly lower than that of GuiHeFeng, and there was no significant difference in plant height between GuiHeFeng and the other six cultivars (Figure 1B). Just as in the case of plant height, only the panicle length (PL) of NaXiangSiMiao and GuiYaXiang was significantly higher and lower than that of GuiHeFeng, respectively, and there was no significant difference in the PL between GuiHeFeng and the other seven cultivars (Figure 1C). There was no significant difference in the number of unfilled grains (NUG) or the number of filled grains (NFG) between GuiHeFeng and all the other nine derivatives (Figure 1D,E). The seed setting rate (SSR) of JingYouXiang139, NaXiangSi and NaGuXiang was significantly higher than that of GuiHeFeng, and there was no significant difference in the SSR between GuiHeFeng and the other six cultivars (Figure 1F). Only the length of flag leaf of NaFengZhan and GuiNongFeng was significantly higher than that of GuiHeFeng, with no significant difference between GuiHeFeng and the other seven cultivars (Figure 1G). Except for HeFengDao445, GuiNongFeng and NaXiangSiMiao, there was no significant difference in the width of flag leaf (WFL) between GuiHeFeng and the other five cultivars (Figure 1H). There was no significant difference in the seed weight per plant (SWPP) between GuiHeFeng and all the other nine derivatives (Figure 1I). There was no significant difference in the thousand-kernel weight (TKW) between GuiHeFeng and the other four cultivars, including HeFengDao445, JingYouXiang139, GuiYaXiang and NaXiangSiMiao (Figure 1J). Ratio of length and width (RLW) of GuiHeFeng and GuiYaXiang was significantly lower than that of other eight cultivars (Figure 1K).

In addition, for the NUG, NFG and SWPP, there was no significant difference between GuiHeFeng and all its nine derivatives; for the PH, PL and LFL, there was no significant difference between GuiHeFeng and seven cultivars; for the EPN and SSR, the number was six cultivars; for the WFL and TKW, the number was five cultivars and fourcultivars, respectively. Over half of the derivatives closely resembled GuiHeFeng in the majority of the agronomic characteristics that were evaluated.

### 2.2. Sequence and SNPs Information Was Produced by Whole-Genome Resequencing

GuiHeFeng and all its nine derivatives were used to carry out whole-genome resequencing to get the basic sequence and SNPs information for further analysis. A total of 751.32 million (M) clean reads of 150 bp that included 111.18 GB of data were generated from the 10 rice varieties, with more than 19× depth (Table 2). More than 98% of the clean reads of 150 bp were mapped to the Nipponbare genome, with the average coverage ratio ranging from 82.42% to 89.69% (Table 2).

**Figure 2:**
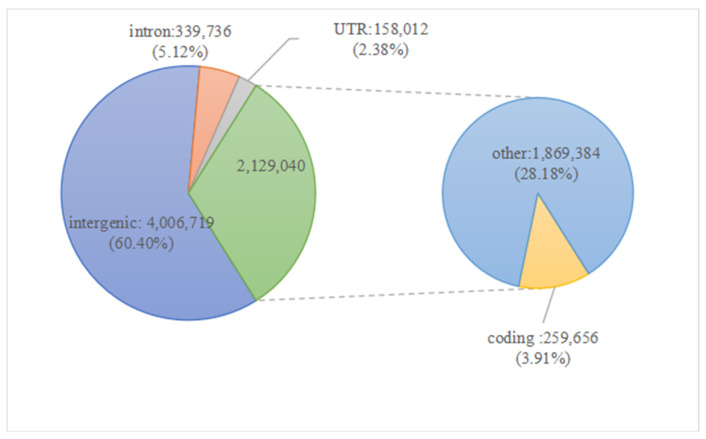
Distribution of SNPs localization for the 10 cultivars tested.

We used GATK v4.0 to call the SNPs [21]. Overall, 6,633,507 SNPs were identified in these MSU 7.0listed 10 cultivars. According to the genome annotation (MSU 7.0), 60.40% of all SNPs were found in intergenic regions, 5.12% in introns, 2.38% in UTRs, 3.91% in gene-coding regions, and 28.18% in other regions (Figure 2; Table S2).

### 2.3. Key GuiHeFeng Traceable Blocks Were Found in the Genomes of Its Derivatives

**Figure 3:**
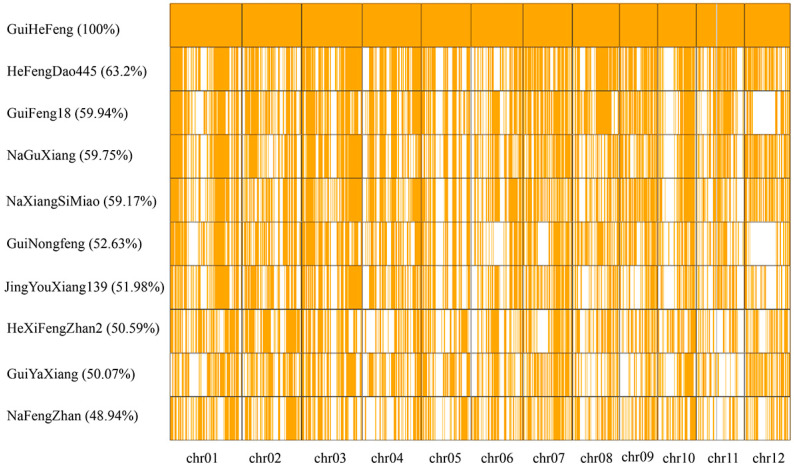
Distribution of GuiHeFeng traceable blocks (GTBs) in the 9 derivatives. The GTBs are represented in orange. The derivative name and the percent of GTBs are shown on the left side.

**Figure 4:**
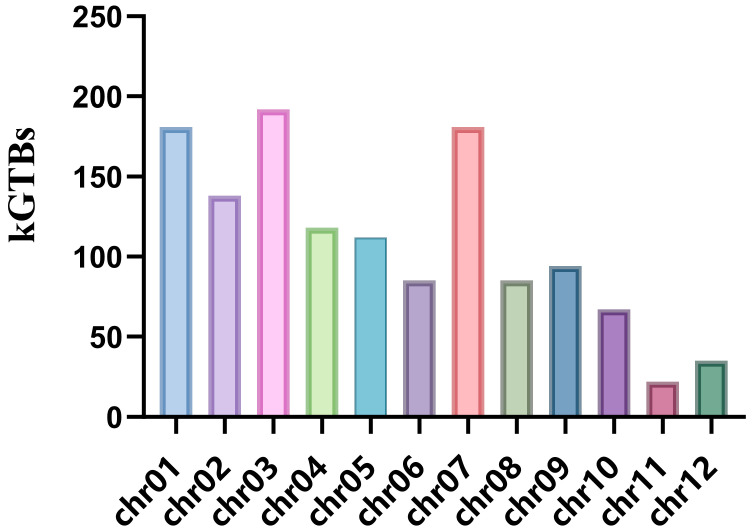
Distribution of the kGTBs across the rice genome. These kGTBs exhibited a non-uniform genomic distribution across the rice genome (chr.1: 181; chr.2: 138; chr.3: 192; chr.4: 118; chr.5: 112; chr.6: 85; chr.7: 181; chr.8: 85; chr.9: 94; chr.10: 67; chr.11: 22; chr.12: 35).

According to the method described by Zhou et al. [5], the rice genome was segmented into 7471 adjacent blocks with a bin size of 50 kb (Table S3). Using a cut-off of more than 85% identity between GuiHeFeng and its derivatives, we delineated the GuiHeFeng traceable blocks (GTBs). As shown in Figure 3, 63.2% of the genomic blocks in HeFengDao445 were identified as GTBs, followed by GuiFeng18 (59.94%), NaGuXiang (59.73%), NaXiangSiMiao (59.17%), GuiNongFeng (52.63%), JingYouXiang139 (51.98%), HeXiFengZhan2 (50.59%), GuiYaXiang (50.07%) and NaFengZhan (48.94%). There were 1310 key GTBs (kGTBs), which were derived from GuiHeFeng and found in all nine derivatives (Table S4). These key GTBs were unevenly distributed on the whole genome of rice, chromosome 3 with the largest number of 192, and chromosome 11 with the lowest number of 22 (Figure 4).

### 2.4. Key Genomic Regions Were Selected from kGTBs and Selection Sweeps

**Figure 5:**
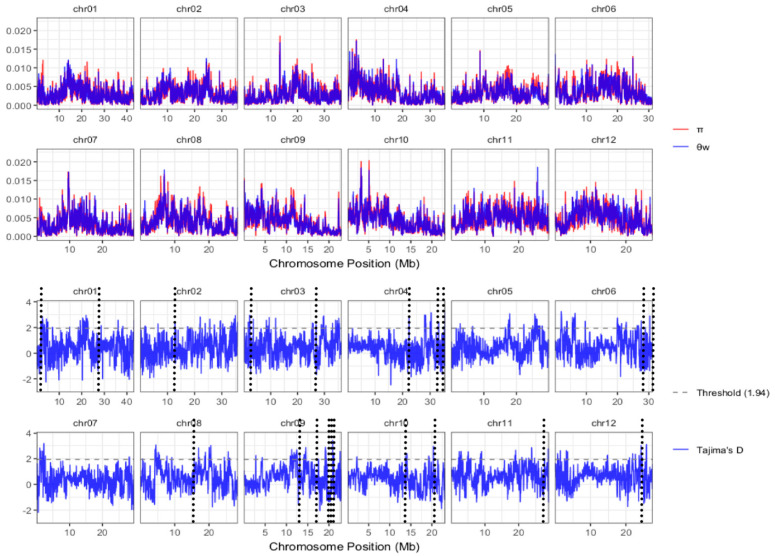
Distribution of SSWs on chromosomes. π (red), θw (deep blue) and Tajima’s D (light blue) are calculated with sliding window of 50 kb across one chromosome. Horizontal black dashed lines indicate threshold (1.94) and vertical black dashed lines indicate kGRs listed in Table S6. In the 20.9-22.7 Mb interval of chromosome 9, we detect three adjacent bins-bin15: 20.90-20.95 Mb, bin16: 21.15-21.20 Mb, and bin17: 22.65-22.70 Mb-indicated by black dashed lines.

Selective sweeps are genomic regions that probably contain excellent alleles relevant to important agronomic traits and are preferably selected by breeder selective sweeps (SSWs) [11]. To exploit the selective sweeps (SSWs) of GuiHeFeng and its derivatives, the π, θw and Tajima’s D [22] were calculated with a sliding window of 50 kb across 12 chromosomes with Variscan [23], with a cut-off of 5% for Tajima’s D test (Tajima’s D ≥ 1.94). We found 375 SSWs, totaling 18.75 Mb, distributed on all chromosomes (Figure 5; Table S5). Furthermore, we found that only 20 SSWs were included in the kGTBs, which were regarded as key genomic regions (kGRs, Table S6). All these 20 kGRs are important for rice breeding and were preferably selected by different breeders.

### 2.5. Excellent Alleles Were Exploited from kGTBs and Key Genomic Regions

Beyond the key genomic regions, rice breeders are particularly interested in the superior alleles located within these areas. To find the excellent alleles on the kGTBs, adjacent SNPs with the same segregation pattern were combined to form a marker for an association test with agronomic traits by a PLINK analysis. According to the results (Table 3 and Table S7), 20 alleles, including 17 known genes, were found on the kGTBs: two genes, *Rd* and *OsCYP704A3*, are associated with seed morphology, two genes, *D2* and *TAC1*, are linked to plant architecture; six genes (*Gnla*, *Rf3*, *OsLG3*, *DPL2*, *GLW7* and *HSA1b*) are related to yield; two genes, *Hd7* and *Hd1*, are involved in the heading date; four genes (*BET1*, *OsJAZ1*, *bZIP73* and *LHCB5*) are related to for biotic stress; and one gene, *OsUGT707A2*, is related to secondary metabolism. The largest number of genes are involved in yield regulation, while only one gene is related to secondary metabolism. The SNPs consistency between GuiHeFeng and all the derivatives of the 17 genes were reconfirmed by a gene chip analysis (Table S8-1). However, the derivatives showed differences from GuiHeFeng at some genes, such as *ALK*, *Badh2* and *Rf2* (Table S8-2). We found no important genes on the 20 key genomic regions by the PLINK analysis. So, we directly found the loci for the key genomic regions (kGRs) in Nipponbare genome IRGSP-1.0 on the Rice Gene Index (RGI; https://riceome.hzau.edu.cn) (accessed on 26 July 2025) platform. As shown in Table 4 and Table S5, there are 38 genes in the key genomic regions, except the key genomic regions on chr.12. To our surprise, among the 38 known genes, 29 genes are involved in defense responses against biotic/abiotic stress, four genes for fertility, only one gene for yield components, and four genes for other functions (Table 4 and Table S9).

## 3. Discussion

### 3.1. Important Genes Were Identified from GuiHeFeng and Its Derivatives

The genes relevant to critical agronomic traits play an important role in rice breeding. For example, the ‘Green Revolution’ gene *sd1* has been used to develop a lot of rice cultivars and has made a significant contribution to increases in rice yields [24]. Exploiting and utilizing important genes from elite germplasm is the permanent target for rice breeders. Important genes, such as *Xa21* [25], *Gn1a* [26], *Wx* [27], *GS5* [28] and *IPA1* [29] for resistance, grain yield, quality and plant type, were identified in the elite rice HuangHuaZhan through whole-genome sequencing and a pedigree analysis [5]. The important gene *TAC1* [30] was also found in HuangHuaZhan [6]. Six important genes, *sd1* [31], *LP* [32], *GW5* [33], *BC10* [34], *RL14* [35] and *OsNAC6* [36], were found in another elite rice, 9311 [19]. We identified 17 important genes in the kGTBs, which exist in both GuiHeFeng and the other nine derivatives (Table 3). Among these 17 genes, two genes, *Gn1a* and *TAC1*, were also found in the seven genes identified in HuangHuaZhan. *Gn1a*, the first major QTL implicated in grain number regulation per panicle, explained 44% of the phenotypic variance [26]. *TAC1* is a major quantitative trait locus, positively controlling the tiller angle in rice [30]. *D2*, identified in GuiHeFeng, also takes an important role in the regulation of tiller angle [37]. It seems that the grain number per panicle and tiller angle, controlled by *Gn1a* and *TAC1*/*D2*, are among the most critical agronomic traits for rice breeders during breeding selection. *Rd* controls the red coat of seeds [38], and *CYP704A3* negatively controls the length of rice seeds [39]. *Hd1* [40] and *DTH2* [41] can both delay the heading date under long-day conditions. A longer heading date results in more biomass and higher yield. Maybe this explains the preference of breeders for *Hd1* and *DTH2* in rice breeding practice. *GLW7* increases both the length and weight of rice grains [42]. Three seed production genes were found in GuiHeFeng and all its derivatives. *Rf3* [43] positively regulates the restoration of fertility, but *DPL2* [44] and *HSA1b* [45] both control hybrid incompatibility. The function of *Rf3* contradicts the function of *DPL2* and *HSA1b*. However, GuiHeFeng and all its derivatives had high seed-setting rates, ranging from 80.5% to 88.2% (Table S1). Moreover, GuiHeFeng showed strong compatibility for both two-line and three-line male sterile lines. For example, the hybrid lines from the cross between GuiHeFeng and the three-line male sterile line SiFeng A and the cross between GuiHeFeng and the two-line male sterile line ChangS showed elite agronomic performance (data unpublished). More research needs to be carried out to illustrate the seed reproduction mechanism of these three genes in GuiHeFeng and its derivatives.

Previous research has showed that biotic and abiotic stress-related genes are favored by breeders [5,6]. Our results are consentaneous with these previous findings. Among the 17 genes, five are stress-related genes, *OsLG3* [46], *BET1* [47], *OsJAZ1* [48], *bZIP73* [49], and *LHCB5* [50]. In addition, among the 38 genes in the key genomic regions, 29 genes are involved in defense responses against biotic/abiotic stress (Table 4). Our results support the following proposal: to highly maintain the breeders, the yield and good quality of the target cultivars wherever cultivated, stress-related genes are spontaneously selected by different breeders to respond to varied environments in rice breeding.

### 3.2. kGTBs and Key Genomic Region Are Useful for Modern Rice Breeding

Marker-assisted selection (MAS) has been successfully utilized to pyramid the elite alleles of important genes, improving the yield, quality and resistance of rice cultivars [51,52]. It is critical to assess the performance of a target allele before its utilization in MAS. Due to uncertainty regarding genetic backgrounds and growing environments, it is difficult to detect the minor-effect QTLs, especially for the abiotic stress-related QTLs, by the traditional QTL analysis method [53,54]. A method named Meta-QTL analysis has been invented to detect the key genomic region, which contains the target allele and is stably inherited in different genetic backgrounds and growing environments [9]. The emergence of high-throughput genome sequencing in combination with the availability of pedigree analysis makes the findings on such key genomic regions more precise and efficient, and a key genomic region related to important agronomic traits been found in rice backbone parents, such as Minghui63, Huanghuazhan, Shuhui527 and Jiayu253 [5,18,19,20]. In the present study, 1310 key GuiHeFeng traceable blocks (Table S4), 375 selective sweeps, and 20 key genomic regions (Table S5) were identified in GuiHeFeng and its derivatives. Moreover, 17 important genes were found on the kGTBs (Table 3), and 38 were found on the 20 key genomic regions (Table 4). The genetic effects of these key genomic regions should be characterized in more detail, and the key genes along with the functional markers should be specified to accelerate MAS selection in rice breeding. These key genomic regions could be used as important blocks for genomic selection (GS) in the future of rice breeding.

Some important genes, for example, most NLR genes, are positionally clustered in a genomic region [13]. Some abiotic stress-related QTLs are also clustered in a genomic region [55,56]. We found three alleles of *Gn1a* clustered on chr.1 and two alleles of *OsLG3* clustered on chr.3 (Table 3). As shown in Table 4, four abiotic stress-related genes, *OsABA1*, *OsAP37*, *OsPT17* and *OsPP65*, were clustered on chr.4; two resistance-related genes, *OsWAK54* and *OsWAK55*, on chr.4; two resistance-related genes, *OsRRK1* and *OsLRR-RLK1*, on chr.4 and 6; meanwhile, nine abiotic stress-related genes were clustered on chr.9. This suggests that alleles of the same gene or QTLs, as well as genes/QTLs with similar functions, frequently cluster within specific genomic regions. In comparison with handling and utilizing individual alleles of genes/QTLs, the key genomic regions that contain clusters of multiple elite alleles demonstrate greater effectiveness in rice breeding, particularly for minor-effect QTLs.

### 3.3. GuiHeFeng Is a Backbone Parent for Rice Breeding

Backbone parents, as carriers of multiple beneficial agronomic traits, are critical for rice breeding [18]. GuiHeFeng is typically a high-yield rice cultivar, showing an increase in yield of 12.32% in comparison with control cultivars. So, it has been used widely by different breeders to develop new rice cultivars. The percentage of GuiHeFeng traceable blocks in the derivatives ranged from 48.94% to 63.20%. However, more than half of the derivatives closely resembled GuiHeFeng in the majority of the agronomic traits that were evaluated (Figure 1). In addition, no derivative showed a significant increase in the seed weight per plant (SWPP) from that of GuiHeFeng. The results indicate that GuiHeFeng is dominant in a large number of yield-related genes/QTLs, showing high heritability in the yield performance. In addition to maintaining the high-yield performance of GuiHeFeng, four derivatives, GuiNongFeng, NaXiangSiMiao, GuiYaXiang and JingYouXiang139, showed an improvement in quality with fragrance Badh2 (Table S8-1). It is feasible to use GuiHeFeng as a high-yield backbone parent, crossing with another unique parent to improve the quantity or resistance of a rice cultivar.

Currently, biotic and abiotic stress tolerance has become a primary objective for rice breeding programs [6]. Our results show that 18 of the 20 key genomic regions, which were identified from GuiHeFeng, contain more than one biotic or abiotic resistance-related gene (Table S8). The results indicate that GuiHeFeng could be used as a stress-resistance parent to develop new high-yield varieties of rice with resistance to stressful environments and unpredictable climate changes.

## 4. Materials and Methods

### 4.1. Plant Materials

A total of 10 rice varieties were used for the analysis in this study (Table 1). GuiHeFeng was one of the two parents of the other 9 derivatives. HeFengDao445 and HeXiFengZhan2Hao were collected from Hechi Agricultural Science Research Institute (Hechi, China); JingYouXiang139 from Guangxi Boshiyuan Seed Industry Co., Ltd. (Nannning, China); and GuiHeFeng and 6 derivatives from the Rice Research Institute of Guangxi Academy of Agricultural Sciences (Nannning, China). All the varieties were planted in the experimental field of the Rice Research Institute, Guangxi Academy of Agricultural Sciences, Nanning (108.22′ E, 22.48′ N), China, in the early season and late season of 2024, and the early season of 2025. The key meteorological data for the entire growth periods were systematically collected as follows: early rice season of 2024 (March–July)—mean temperature 26.5 °C, cumulative precipitation 680 mm; late rice season of 2024 (July–November)—mean temperature 28.2 °C, cumulative precipitation 410 mm; early rice season of 2025 (March–July)—mean temperature 26.6 °C, cumulative precipitation 610 mm. Each variety was planted in three plots, with 5 rows in each plot and 10 plants in each plot. The spacing between the plants and plots was 20 cm × 20 cm and 30 cm × 30 cm, respectively. The plots of all the varieties were arranged in a randomized complete block design.

### 4.2. Agronomic Trait Investigation

A randomized complete block design was adopted. To avoid border effects, five healthy plants free from diseases, pests, and lodging were randomly selected from the inner area of each plot. The number of effective tillers per plant was recorded at the peak tillering stage. At maturity, the following agronomic traits were measured: plant height (PH), effective panicle number (EPN), panicle length (PL), length of flag leaf (LFL) and width of flag leaf (WFL). The plants were harvested individually and threshed manually. The grain-related traits, including the number of filled grains (NFG), number of unfilled grains (NUG), seed weight per plant (SWPP), thousand-kernel weight (TKW), and grain length-to-width ratio (RLW), were determined using a grain appearance quality analyzer (SC-E, Hangzhou Wanshen Detection Technology Co., Ltd., Hangzhou, China). The seed-setting rate (SSR) was calculated as the percentage of filled grains to the total spikelets. All the data were expressed as the mean values from the three plots (15 plants in total) from the statistical analysis.

### 4.3. Genome Resequencing and SNP Calling

A single individual of each variety was selected for whole-genome resequencing. The genomic DNA was extracted from young leaves using a DNA Extraction Kit (Qiagen, Hilden, Germany), sequenced on an Illumina X10 platform (150 bp reads and 300–500 bp insert). We removed the low-quality paired reads (including those with putative PCR duplicates, with >10 nucleotides aligned to the adapter, with ≥10% unidentified nucleotides (N) and >50% bases having Phred quality < 10) [57]. The clean reads were mapped to the reference genome of Nipponbare (MSU v7.0) by using Burrows–Wheeler Alignment (BWA) software (v0.7.12) [58]. The sequencing depth, genome coverage, and other information for each sample were calculated by SAMtools (version 1.2) software [59]. The SNPs were identified using GATK (v4.0) [21] and annotated with SnpEff (v4.1) [60].

### 4.4. Construction of Genome Bins and Identification of Key Genomic Region and Selection Sweep Region

The genome was segmented into non-overlapping bins of 50 kb length. The similarity between each sample of the 9 derivatives and GuiHeFeng was calculated to obtain the similarity matrix for each bin. If the identity of the bin of the tested derivative and GuiHeFeng was larger than or equal to 0.85, then it was deemed a conserved block (GuiHeFeng traceable block, GTB). Such a GTB found in all 9 derivatives was considered a key GTB (kGTB). To identify the selection sweeps (SSWs), the θw and Tajima’s D [22] were calculated with a sliding window of 50 kb across 12 chromosomes with Variscan [23], using the SNPs identified from resequencing. We used 5% as a cut-off for Tajima’s D test (Tajima’s D ≥ 1.94) to identify the top selective sweeps with high significance. The regions found in both the kGTBs and top selective sweeps were identified as key genomic regions (kGRs) for rice breeding. Figures of the key blocks or selection sweep regions were drawn using Perl script with GD module (www.perl.org) (accessed on 26 September 2025).

### 4.5. Association Test and Gene Chip Analysis

For the kGTBs, adjacent SNPs with the same segregation pattern were combined to form a marker for an association test [5]. PLINK was used to analyze the association between these markers and 11 agronomic traits in a linear model [61]. The important loci for agronomic traits were determined as those with FDR *p*-values less than 0.0001 from 100,000 permutation tests. The important loci for the key genomic regions (kGRs) were found in the Nipponbare genome IRGSP-1.0 on the Rice Gene Index (RGI; https://riceome.hzau.edu.cn) (accessed on 26 July 2025) platform. The whole-genome SNP array GSR40K was employed to analyze the variations in 164 functional genes. The GSR40K analysis was performed at Wuhan Greenfafa Institute of Novel Gene chip R&D Co., LTD (Wuhan, China) (https://www.greenfafa.com/) (accessed on 20 August 2025), according to the Infinium HD Assay Ultra Protocol (HYPERLINK: https://www.illumina.com) (accessed on 12 September 2025).

## 5. Conclusions

Through an in-depth analysis of key genomic regions in Guifeng rice using SNP data, this study integrated kGTB and SSW strategies to pinpoint the critical genomic regions and identify the superior alleles. It reveals the genetic basis of yield-related traits and key genomic regions underlying stable inheritance in GuiHeFeng rice. These findings provide new insights for advancing molecular design, breeding and genomic selection in GuiHeFeng rice.

## Figures and Tables

**Table 1:** Origin and pedigree of GuiHeFeng and its derivatives.

No.	Variety	Pedigree	Cultivar Developer	Generation
1	GuiHeFeng	HeFengZhan/YueTaiZhan	Rice Research Institute, Guangxi Academy of Agricultural Sciences (Nanning, China)	F_9_
2	GuiFeng18	GuiHeFeng/MeiXiangZhan	Rice Research Institute, Guangxi Academy of Agricultural Sciences (Nanning, China)	F_8_
3	HeFengDao445	GuiHeFeng/GuiYu9Hao	Hechi Agricultural Science Research Institute, Hechi Branch, Guangxi Academy of Agricultural Sciences (Hechi, China)	F_9_
4	NaFengZhan	GuiHeFeng/ZaoHui3Hao/GuiHui1561	Rice Research Institute, Guangxi Academy of Agricultural Sciences (Nanning, China)	F_8_
5	JingYouXiang139	BaiXiang139/GuiHeFeng	Guangxi Boshiyuan Seed Industry Co., Ltd. (Nanning, China)	F_9_
6	GuiYaXiang	GuiHeFeng/XiangChangMang	Rice Research Institute, Guangxi Academy of Agricultural Sciences (Nanning, China)	F_8_
7	GuiNongFeng	GuiHeFeng/YeXiangZhan	Rice Research Institute, Guangxi Academy of Agricultural Sciencesm(Nanning, China)	F_9_
8	NaXiangSiMiao	GuiHeFeng/BaiXiang139/GuiHui110	Rice Research Institute, Guangxi Academy of Agricultural Sciencesm(Nanning, China)	F_8_
9	NaGuXiang	GuiHeFeng/BaiXiang139/HuangHuaZhan	Rice Research Institute, Guangxi Academy of Agricultural Sciencesm (Nanning, China)	F_9_
10	HeXiFengZhan2Hao	HeXiXiang/GuiHeFeng	Hechi Agricultural Science Research Institute, Western Guangxi Branch, Guangxi Academy of Agricultural Sciences (Hechi, China)	F_8_

**Table 2:** Resequencing of GuiHeFeng and its 9 derivatives.

Variety	Reads (M)	Bases (G)	Map Reads (%)	Map Reads	Depth X	Cov_ratio (%)
GuiHeFeng	124.74	18.59	98.59	122,978,517	51.78	89.69
GuiFeng18	58.54	8.72	98.73	57,794,774	24.51	85.53
HeFengDao445	66.84	9.93	98.77	66,016,096	27.99	86.67
NaFengZhan	70.66	10.53	98.62	69,682,802	29.65	86.98
JingYouXiang139	84.91	12.60	98.70	83,805,328	35.4	87.92
GuiYaXiang	76.20	11.34	98.58	75,121,141	31.73	87.22
GuiNongFeng	71.49	10.66	98.61	70,495,489	29.83	87.66
NaXiangSiMiao	81.89	12.17	98.62	80,756,694	34.09	87.74
NaGuXiang	68.9	10.23	98.62	67,954,951	28.74	86.36
HeXiFengZhan2	47.15	7.04	98.46	46,426,567	19.98	82.42
Sum	751.32	111.18				

**Table 3:** Important alleles relevant to agronomic traits on kGTBs.

No.	Chr	Start	End	Gene	Function	Category	Gene Chip Result
1	chr01	25,383,093	25,383,093	*Rd*/*DFR*/*OsDfr*	red seed coat	Seed morphology	T ^1^
2	chr01	5,244,076	5,244,076	*D2*/*CYP90D2*/*SMG11*	larger tiller angle	Plant architecture	T
3	chr01	5,270,928	5,270,928	*Gn1a*/*OsCKX2*	increasing grain number	Yield components	T
4	chr01	5,275,530	5,275,530	*Gn1a*/*OsCKX2*	increasing grain number	Yield components	T
5	chr01	5,275,544	5,275,544	*Gn1a*/*OsCKX2*	increasing grain number	Yield components	T
6	chr01	5,568,692	5,568,692	*Rf3*/*OsMADS3*	fertility restoration	Yield components	T
7	chr02	30,096,330	30,096,330	*DTH2*/*Hd7*	delaying heading date under LD	Heading date	T
8	chr03	4,353,347	4,353,347	*OsLG3*	increasing drought tolerance	Yield components	T
9	chr03	4,353,103	4,353,103	*OsLG3*	increasing drought tolerance	Yield components	T
10	chr04	23,886,659	23,886,659	*BET1*	increasing boron toxicity tolerance	Abiotic stress	T
11	chr04	28,894,753	28,894,753	*OsCYP704A3*	longer seed size	Seed morphology	T
12	chr04	33,304,910	33,304,910	*OsJAZ1*	decreasing root length and weight	Abiotic stress	T
13	chr06	4,201,227	4,201,227	*DPL2*	hybrid incompatibility	Yield components	T
14	chr06	9,338,220	9,338,220	*Hd1*	promoting heading date under LD	Heading date	T
15	chr07	19,060,398	19,060,398	*OsUGT707A2*	more 5-O-glucoside	Secondary metabolism	T
16	chr07	19,103,249	19,103,249	*OsSPL13*/*GLW7*	increasing grain size	Yield components	T
17	chr09	18,122,850	18,122,850	*bZIP73*	decreasing chilling tolerance	Abiotic stress	T
18	chr09	20,731,844	20,731,844	*TAC1*	spread-out plant architecture	Plant architecture	T
19	chr11	7,659,694	7,659,694	*LHCB5*	increasing blast resistance	Biotic stress	T
20	chr12	24,669,797	24,669,797	*HSA1b*	hybrid incompatibility	Yield components	T

^1^ Note: T is the abbreviation for true, meaning this gene/QTL exhibits a consistent SNP genotype between GuiHeFeng and the other varieties. This result was validated using a gene chip.

**Table 4:** Important alleles relevant to agronomic traits on key genomic regions (kGRs).

No.	Chr	Start	End	Gene	Function	Category
1	chr01	2,053,583	2,057,638	LRK10L-2.1	Resistance gene analogs (RGAs)	Biotic stress
2	chr01	2,8666,309	28,668,106	*Xa21*	Bacterial blight resistance	Biotic stress
3	chr01	28,669,479	28,673,568	*OsLRR-RLK*	Regulates defense reaction	Biotic stress
4	chr02	12,798,344	12,804,729	Retrovirus-related Pol polyprotein from transposon RE1	Increases resistance to broad bean wilt virus 2	Biotic stress
5	chr03	26,952,048	26,959,200	*OsTHIC*	Positively REGULATE vitamin B 1 synthesis	Other
6	chr03	3,489,869	3,500,130	*TOP3α*	Regulates meiotic recombination	Other
7	chr04	22,369,632	22,376,812	*OsABA1*	Positively regulates plant development and adaptation to abiotic and biotic stresses	Biotic/abiotic stress
8	chr04	22,353,707	22,355,207	*OsAP37*	Mediates tolerance to drought	Abiotic stress
9	chr04	22,362,239	22,367,204	*OsPT17*	Involved in chilling response and salt stress	Abiotic stress
10	chr04	22,389,303	22,393,831	*OsPP65*	Decreases rice resistance to chilling	Abiotic stress
11	chr04	33,185,813	33,186,889	*OsWAK54*	Plays important roles in cell expansion and pathogen resistance	Biotic stress
12	chr04	33,192,623	33,196,131	*OsWAK55*	Plays important roles in cell expansion and pathogen resistance	Biotic stress
13	chr04	35,287,781	35,289,156	*OsPR5*	Increases pathogen resistance	Biotic stress
14	chr04	35,270,952	35,276,805	*OsSPARK2*	Negatively regulates tolerance	Biotic/abiotic stress
15	chr06	28,941,271	28,943,704	*OsRRK1*	Positively regulates brown planthopper resistance	Biotic stress
16	chr06	28,905,577	28,909,089	*OsLRR-RLK1*	Initiates striped stem borer resistance	Biotic stress
17	chr06	30,357,699	30,361,201	*OsNPSN11*	Positively regulates blast resistance	Biotic stress
18	chr08	15,695,534	15,703,960	Protein PHR1-LIKE 3	Enhances tolerance to Pi deficiency and salt stress in rice	Abiotic stress
19	chr09	131,54,943	13,155,832	*OsSAP17*	Enhances plant resistance to drought and salt	Abiotic stress
20	chr09	13,181,330	13,184,741	*OsPHD38*	Mediates tolerance to drought and salt stress	Abiotic stress
21	chr09	17,556,929	17,558,591	*OsDjC69*	Mediates flowering and tolerance to drought and salt stress	Abiotic stress
22	chr09	17,565,471	17,566,197	*OsbHLH043*	Mediates tolerance to drought and arsenic stress	Abiotic stress
23	chr09	20,915,301	20,919,808	*OsMYB85*	Cell wall regulators	Other
24	chr09	21,151,736	21,154,358	*OsCYP-24*	Mediates tolerance to drought and salt stress	Abiotic stress
25	chr09	21,155,956	21,157,945	*OsRNS4*	Enhanced tolerance to high salinity	Abiotic stress
26	chr09	21,171,653	21,174,067	*OsPAD1*	Regulates pollen aperture formation	Fertility
27	chr09	21,189,381	21,190,738	*OsMYB31*	Increases yield	Yield components
28	chr09	21,197,503	21,199,723	*MS5*	Regulates pollen formation	Fertility
29	chr09	21,199,731	21,202,763	*OsAPX9*	Increases tolerance to drought, plant height and heading date	Abiotic stress/heading date/plant Architecture
30	chr09	22,653,849	22,657,046	*Ohp2*	Positively mediates tolerance to salt stress	Abiotic stress
31	chr09	22,666,306	22,671,392	*OsWD40-174*	Has important role in rice–*Xoo* interactions	Biotic stress
32	chr10	20,355,076	20,355,657	*OsERF18*	Enhances tolerance to Pi deficiency	Abiotic stress
33	chr10	20,374,252	20,375,455	*OsEMSA1*	Involved in embryo sac development	Fertility
34	chr10	20,377,195	20,380,235	*OsNP1*	Required for another cuticle formation and pollen exine patterning	Fertility
35	chr10	20,377,031	20,386,096	*OsPLDbeta1*	Activates defense responses and increases disease resistance in rice	Biotic stress
36	chr11	28,804,248	28,808,550	*OsHSP70*	Induces tolerance to high-temperature stress	Abiotic stress
37	chr11	28,827,676	28,828,513	*OsMT1a*	Positively regulates rice resistance to blast	Biotic stress
38	chr11	28,845,905	28,852,938	*OsSCL57*	Regulates the phosphorus homeostasis of rice	Other

## Data Availability

The original contributions presented in this study are included in the article. Further inquiries can be directed to the corresponding authors.

## Supplementary Materials

The following supporting information can be downloaded at: https://www.mdpi.com/article/10.3390/plants15030520/s1, Table S1: The mean ± SD of 11 agronomic traits investigated over three growth seasons for all cultivars; Table S2: Results of SNPs annotation; Table S3: All bins from GuiHeFeng matrix; Table S4: Important genes on the kGTBs; Table S5: Selective sweeps (SSWs); Table S6: Key genomic regions (kGRs); Table S7: Important genes on the kGTBs; Table S8-1: Genotype of 164 genes/QTLs detected by GSR40K analysis of GuiHeFeng and its derivatives; Table S8-2: Genes with different genotypes between GuiHeFeng and its derivatives; Table S9: Important genes on kGRs.
