# Supplementary material for: Key Genomic Regions of Rice Cultivar GuiHeFeng and Its Derivatives Revealed by Genome-Wide Analysis
Source: Plants (Basel). 2026 Feb 6;15(3):520. doi: 10.3390/plants15030520 (PMC12899404; doi:10.3390/plants15030520)
Supplement: Supplementary file 1 [file plants-15-00520-s001.zip › Table S2 Results of SNPs annotation.pdf]

Table S2. Results of SNPs annotation.

Region	Type	Num
--	INTERGENIC	4006719
--	INTRON	339736
--	UPSTREAM	1188190
--	DOWNSTREAM	670594
--	UTR_5_PRIME	51861
--	UTR_3_PRIME	106151
--	SPLICE_SITE_ACCEPTOR	444
--	SPLICE_SITE_DONOR	433
--	START_GAINED	9649
--	TRANSCRIPT	73
--	GENE_FUSION	1
CDS	START_LOST	317
CDS	FRAME_SHIFT	829
CDS	SYNONYMOUS_START	1
CDS	NON_SYNONYMOUS_START	37
CDS	CODON_INSERTION	18
CDS	CODON_DELETION	13
CDS	EXON_DELETED	1
CDS	SYNONYMOUS_CODING	107969
CDS	CODON_CHANGE_PLUS_CODON_ INSERTION	34
CDS	CODON_CHANGE_PLUS_CODON_ DELETION	22
CDS	NON_SYNONYMOUS_CODING	139769
CDS	SYNONYMOUS_STOP	183
CDS	STOP_GAINED	2689
CDS	STOP_LOST	616
CDS	EXON	7158
--	Other	0
SUM		6633507
